# Activation of the JNK/MAPK Signaling Pathway by TGF-β1 Enhances Neonatal Fc Receptor Expression and IgG Transcytosis

**DOI:** 10.3390/microorganisms9040879

**Published:** 2021-04-20

**Authors:** Shaoju Qian, Chenxi Li, Xi Liu, Xiangchao Jia, Yuncai Xiao, Zili Li

**Affiliations:** 1 State Key Laboratory of Agricultural Microbiology, College of Veterinary Medicine, Huazhong Agricultural University, Wuhan 430070, China; qianshaoju@webmail.hzau.edu.cn (S.Q.); ad630410211@webmail.hzau.edu.cn (C.L.); liuxi1@webmail.hzau.edu.cn (X.L.); jiaxianghcao@webmail.hzau.edu.cn (X.J.); xyc88@mail.hzau.edu.cn (Y.X.); 2 Key Laboratory of Preventive Veterinary Medicine in Hubei Province, The Cooperative Innovation Center for Sustainable Pig Production, Wuhan 430070, China; 3 Key Laboratory of Development of Veterinary Diagnostic Products, Ministry of Agriculture of the People’s Republic of China, Wuhan 430070, China

**Keywords:** TGF-β1, neonatal Fc receptor, JNK pathway, mucosal immunity

## Abstract

The neonatal Fc receptor (FcRn) transports maternal immunoglobulin G (IgG) to the foetus or newborn and protects the IgG from degradation. FcRn is expressed in several porcine tissues and cell types and its expression levels are regulated by immune and inflammatory events. IPEC-J2 cells are porcine intestinal columnar epithelial cells that were isolated from neonatal piglet mid-jejunum. We hypothesized that transforming growth factor β1 (TGF-β1) upregulated pFcRn expression in IPEC-J2 cells. To test this hypothesis, we treated IPEC-J2 cells with TGF-β1 and demonstrated that porcine FcRn (pFcRn) expression was significantly increased. SP600125, a specific mitogen-activated protein kinase (MAPK) inhibitor, reduced TGF-β1-induced pFcRn expression in IPEC-J2 cells. We performed luciferase reporter assays and showed that the c-JUN sensitive region of the pFcRn promoter gene was located between positions −1215 and −140. The c-JUN sequence, in combination with the pFcRn promoter, regulated luciferase reporter activity in response to TGF-β1 stimulation. Chromatin immunoprecipitation confirmed that there were three c-JUN binding sites in the pFcRn promoter. Furthermore, in addition to increased pFcRn expression, TGF-β1 also enhanced IgG transcytosis in IPEC-J2 cells. In summary, our data showed that the modulation of JNK/MAPK signaling by TGF-β1 was sufficient to upregulate pFcRn expression.

## 1. Introduction

The neonatal Fc receptor (FcRn), the specific receptor for immunoglobulin G (IgG), has a similar structure to major histocompatibility complex class I-like biomolecules which consist of covalently linked α heavy and β2M light chains. FcRn is widely expressed on the surface of epithelial cells, macrophages, and dendritic cells [1]. FcRn is involved in the transcellular transport of IgG; for example, FcRn-mediated IgG transport, in the female reproductive tract mucosa, plays an anti-infection role [2]. In addition, FcRn is reported to prevent IgG and albumin degradation during the internalization by endothelial and hematopoietic cells, increasing their half-life [3,4,5]. FcRn is also involved in the cross-presentation of the immune complexes formed by the IgGs and their antigens [6]. Furthermore, Fc fragment fusion proteins can be used as immunogenic antigens to improve vaccine effectiveness [7,8]. FcRn is also reported to participate in immune surveillance, especially in antigen presentation, phagocytosis, and mucosal immunity [6,9,10,11,12,13].

IgG and secretory IgA are the main Ig isotypes present in mucosal secretions, which are actively transported by FcRn and polymeric immunoglobulin receptor (pIgR), respectively. Epithelial cells are placed at the forefront of mucosal immune defence and they secrete a variety of soluble factors, including proteases, oxidants, cytokines/chemokines, and growth factors, which are involved in inflammation and tissue remodelling [14]. Several factors, such as tumour necrosis factor α (TNF-α), interleukin 1β (IL-1β), lipopolysaccharide (LPS), and oxidative stress, activate c-JUN N-terminal kinase (JNK)/AP-1 and NF-κB, resulting in the induction of inflammation [15]. Both pIgR and FcRn are regulated by inflammatory factors, such as IL-1β, interferon (IFN-γ), and TNF-α [16,17,18,19]. Recent studies showed that in human bronchial epithelial cells, transforming growth factor beta 1 (TGF-β1) induced pIgR production in a dose-dependent manner via the p38/mitogen-activated protein kinase (MAPK) signaling pathway [20,21]. Therefore, we hypothesized that TGF-β1 is involved in the regulation of FcRn expression.

TGF-β1 is a multifunctional cytokine that modulates cell growth, differentiation, and migration [22,23]. It also promotes the conversion and reorganization of mucosal plasma cells into the production of IgA, which plays an important role in preventing microbial infection and controlling symbiotic flora in the mucosal tissues [24]. TGF-β1 triggers IgA synthesis and induces its transepithelial transport, further supporting the view that TGF-β1 is one of the key factors involved in mucosal homeostasis.

The MAPK subfamily, comprising three main subfamilies, p38, JNK, and extracellular signal-regulated kinase (ERK), mediates transduction pathways induced by inflammation. Several factors, such as inflammatory mediators, cellular stress, and growth factors, can activate the MAPK signaling pathway [25]. For example, viral infection activates the production of pro-inflammatory factors. TNF-α, in turn, activates the NF-κB signaling pathway to upregulate FcRn expression and enhance IgG transport [18]. Transmissible gastroenteritis virus (TGEV) infection up-regulates the expression of TNF-α, IL-6, IL-8 and TGF-β in PK-15 cells [26]. TGEV infection induces enterotoxigenic *Escherichia coli* K88 (ETEC K88) adhesion by up-regulating the expression of TGF-β in IPEC-J2 cells [27]. We previously reported that TGEV significantly upregulated TGF-β1 secretion, resulting in the induction of pFcRn expression [28]; however, the exact mechanism was not clear. Here, we investigated the molecular mechanisms involved in the upregulation of pFcRn expression by TGF-β1.

## 2. Materials and Methods

### 2.1. Cells and Antibodies

IPEC-J2 cells (generously donated by Dr. Li Xiaoping of Huazhong Agricultural University (Wuhan, China)) were cultured in Dulbecco’s Modified Eagle’s Medium (Hyclone, Beijing, USA) supplemented with 10% fetal bovine serum (Gibco, Waltham, MA, USA), 1% penicillin/streptomycin in an atmosphere of 5% CO_2_ at 37 °C. The affinity-purified rabbit anti-cytoplasmic tails of porcine FcRn polyclonal antibodies were prepared in-house [29]. Horseradish peroxidase-conjugated goat anti-rabbit or goat anti-mouse IgG and the mouse monoclonal anti-glyceraldehyde 3-phosphate dehydrogenase (GAPDH) antibody (mAb) were purchased from ABclonal (Wuhan, China). Rabbit mAbs against phospho-ERK1/2, ERK1/2, phospho-p38, p38, phospho-JNK1/2, JNK1/2, phospho-c-JUN, and c-JUN were obtained from Cell Signaling Technology (Beverly, MA, USA). TGF-β1 was purchased from R&D Systems (Minneapolis, MN, USA).

### 2.2. Western Blotting

Cells were washed twice with cold PBS and incubated on ice with RIPA Lysis Buffer (Beyotime, Shanghai, China) containing protease inhibitor cocktail (Roche, Basel, Switzerland). The cell lysates were prepared and separated with sodium dodecyl sulfate-polyacrylamide gel electrophoresis (SDS-PAGE). The proteins were then transfer-embedded onto a polyvinyl-idene difluoride membrane (Bio-Rad, Richmond, CA, USA). Briefly, proteins were separated using sodium dodecyl sulphate-polyacrylamide gel electrophoresis (12% gels) and then transferred to polyvinylidene difluoride membranes (Bio-Rad). The membranes were blocked with PBST containing 5% skim milk (BD, San Jose, CA, USA) for 1 h and then incubated with the primary antibody overnight at 4 °C, followed by the corresponding horseradish peroxidase (HRP)-conjugated secondary antibody incubation for 1 h. The primary antibodies used here includes the affinity purified rabbit anti-pFcRn-CT polyclonal antibody (1:1000), mouse anti-GAPDH(1:1000), rabbit anti-phospho-ERK1/2 (1:1000), anti-ERK1/2 (1:1000), anti-phospho-p38 (1:1000), anti-p38 (1:1000), anti-phospho-JNK 1/2 (1:1000), anti-JNK1/2 (1:1000), as well as anti-phospho-c-JUN (1:1000) and anti-c-JUN antibody (1:1000). The secondary antibodies used in this step included the goat anti-mouse IgG (1:5000) or goat anti-rabbit IgG antibodies (1:5000). GAPDH was employed as the loading standard. The protein bands were quantified with the ImageJ software. Western blotting analysis was performed as previously described [30].

### 2.3. MAPKs Inhibition Assays

IPEC-J2 cells (70–80% confluence) were treated or untreated with pathway inhibitors (1, 5, 10 μM) SP600125, (1, 5, 10 μM) SB203580, or (1, 5, 10 μM) U0126 (New England Bi-olabs) for 2 h before being stimulated with TGF-β1 (8 ng/mL). The cells were harvested at the indicated time points (12 h) by RIPA Lysis Buffer (Beyotime) containing protease inhibitors cocktail (Roche, Basel, Switzerland). Western blot assays were performed to examine the expression of specified proteins (FcRn, GAPDH, p38, p-p38, ERK, P-ERK, JNK, p-JNK, JUN and c-JUN).

### 2.4. Construction of Reporter Plasmid and Luciferase Assays

The promoter fragment of the pFcRn gene was amplified to construct the luciferase reporter. Luciferase reporter plasmids (F1-9), containing sequences from complete pFcRn promoter or truncated promoter fragment, were constructed by PCR-amplified products (Table 1) into the pGL3 vector (Promega, Madison, WI, USA) through Sac I and Hind III digestion. IPEC-J2 cells (70–80% confluence) were co-transfected with Luciferase reporter plasmid (0.2 μg), together with the pRL-TK plasmid (0.1 μg). Twenty-four hours later, cells were incubated with TGF-β1 (8 ng/mL) for 12 h and their fluorescence was measured via a dual-luciferase enzyme reporter assay system (Promega, Madison, WI, USA) using the manufacturer’s provided protocol. 

### 2.5. Chromatin Immunoprecipitation

The transcription factor binding sites of pFcRn promoter regions were identified by the Transcription Element Search System (TESS). Binding site sequences were analysed by chromatin immunoprecipitation (ChIP) using the manufacturer’s protocol (Beyotime). Briefly, IPEC-J2 cells were treated with or without TGF-β1 for 12 h and fixed with 1% formaldehyde. Next, the nuclei were extracted and the DNA was sheared using ultrasound. Chromatin immunoprecipitation was performed by incubating DNA with 1 μg anti-c-JUN Ab (or 1 μg normal IgG as a negative control) on an orbital shaker at 50–100 rpm for 2 h at room temperature. DNA samples were amplified under optimized conditions using the PCR primers listed in Table 2.

### 2.6. Statistical Analyses

Data from three independent experiments were analysed by one-way analysis of variance using the GraphPad Prism software (version 5.0, GraphPad software, San Diego, CA, USA). Data are presented as the mean ± SD; * *p* < 0.05, ** *p* < 0.01.

## 3. Results

### 3.1. TGF-β1 Upregulated pFcRn Expression in IPEC-J2 Cells

**Figure 1:**
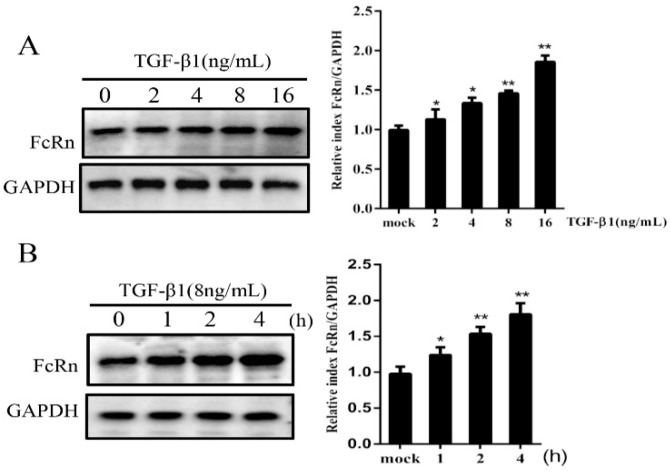
TGF-β1 upregulates pFcRn expression in a dose- and time-dependent manner. (**A**) IPEC-J2 cells were stimulated with TGF-β1 at the indicated dosages (0, 2, 4, 8 and 16 ng/mL) and pFcRn expression was analysed by Western blotting. (**B**) IPEC-J2 cells were incubated with TGF-β1 (8 ng/mL) and collected at 1, 2 and 4 h, followed by the Western blot analysis of pFcRn expression. The right panel represents protein band quantification determined by densitometry and normalized to GAPDH; * *p* < 0.05, ** *p* < 0.01.

First, we evaluated the effect of TGF-β1 on pFcRn protein expression. Western blotting results showed that pFcRn protein expression levels were increased 1.8-fold after 2 h of 16 ng/mL TGF-β1 stimulation compared to control cells (Figure 1A). Furthermore, IPEC-J2 cells treated with TGF-β1 (8 ng/mL) for 2 h and 4 h increased pFcRn protein expression levels by 1.5- and 1.7-fold, respectively (Figure 1B). These results indicated that TGF-β1 increased pFcRn protein expression in a dose- and time-dependent manner.

### 3.2. Effects of MAPK Inhibition on pFcRn Expression

**Figure 2:**
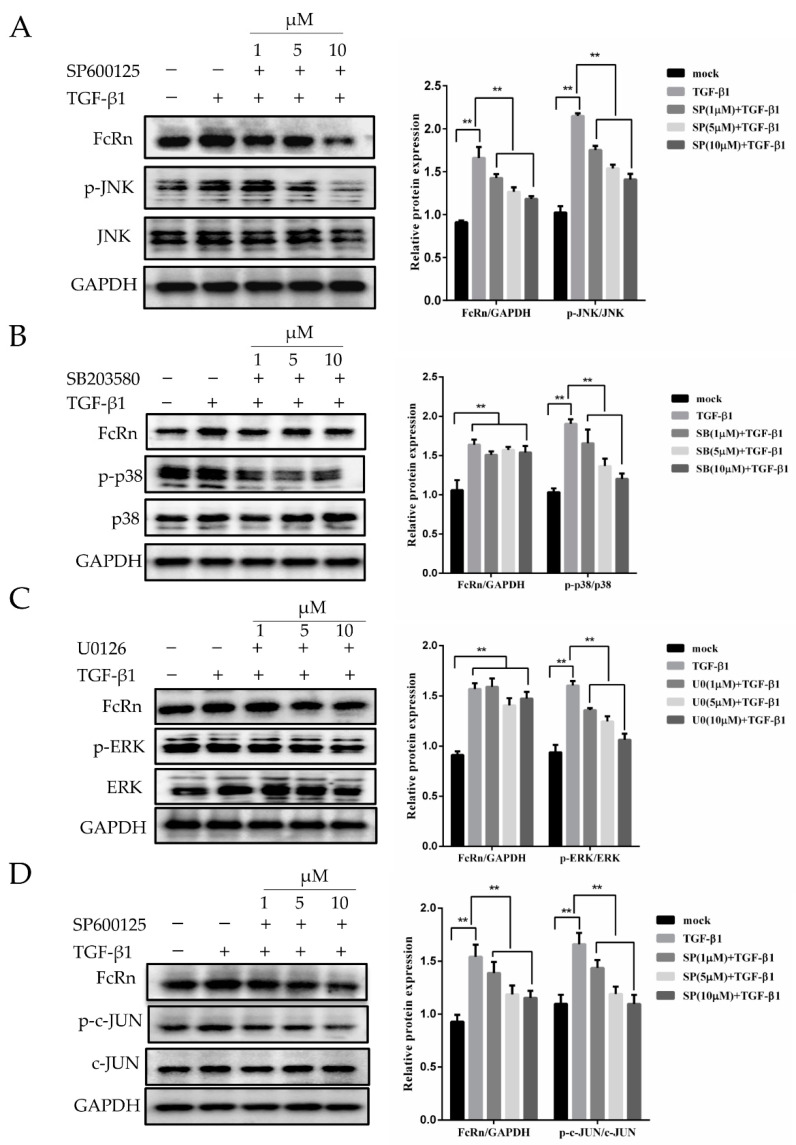
TGF-β1 upregulates pFcRn expression via the JNK/MAPK and c-JUN signaling pathways. (**A**) IPEC-J2 cells were pre-treated with SP600125 (1, 5, and 10 μM), followed by incubation with TGF-β1 (8 ng/mL) for 12 h. IPEC-J2 cells were harvested and Western blotting was performed as described in Section 2. (**B**–**D**) were performed as described in (**A**) using the following inhibitors: p38 inhibitor SB203580 (1, 5 and 10 μM), ERK inhibitor U0126 (1, 5, and 10 μM), and JNK inhibitor SP600125 (1, 5, and 10 μM). GAPDH was used as a loading control. The right panel represents protein band quantification determined by densitometry and normalized to GAPDH, ** *p* < 0.01.

To evaluate JNK, p38, and ERK activation in our model system, cells were pre-treated with SB203580 (p38 inhibitor), SP600125 (JNK1/2 inhibitor) and U0126 (ERK1/2 inhibitor) for 2 h, and then incubated with TGF-β1 (8 ng/mL) for 12 h. We observed that the increasing inhibitor concentrations of MAPK pathway inhibitors reduced the ratios of p-JNK/JNK, p-p38/p38, p-ERK/ERK, and p-c-JUN/c-JUN, while TGF-β1 treatment did not have an effect on the total protein levels of JNK, p38, ERK, and c-JUN (Figure 2). The JNK1/2 inhibitor SP600125 significantly decreased pFcRn expression in a dose-dependent manner, suggesting that the JNK1/2 signaling pathway played a role in TGF-β1-induced pFcRn expression (Figure 2A). However, treatment with SB203580 or U0126 did not affect pFcRn expression, indicating that p38 and ERK MAPK were not involved in the regulation of pFcRn expression in TGF-β1-stimulated IPEC-J2 cells (Figure 2B,C). Furthermore, compared to the TGF-β1-treated group, SP600125 exhibited a reduced ability to upregulate the ratios of the phosphorylated p-c-JUN/c-JUN protein, as well as pFcRn protein (Figure 2D). Further analysis showed that TGF-β1 promoted the phosphorylation of the JNK transcription factor c-JUN, suggesting that TGF-β1 triggered pFcRn expression via the JNK/c-JUN signaling pathway.

### 3.3. Screening for c-JUN Binding Sites Adjacent to the pFcRn Promoter

**Figure 3:**
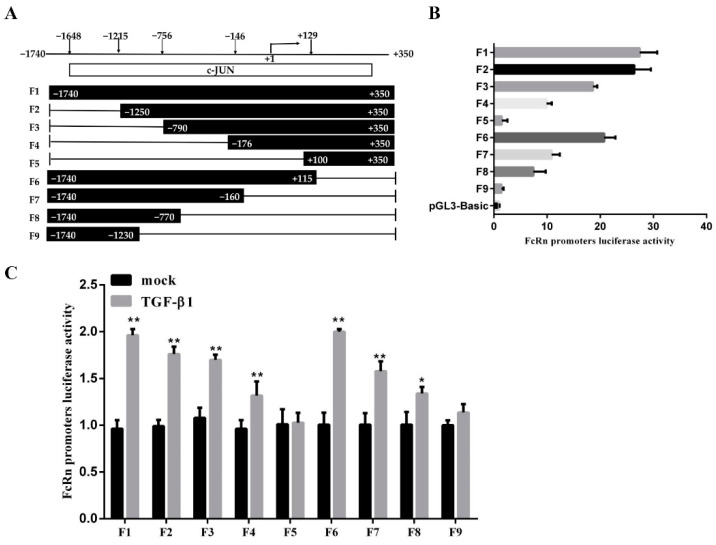
Construction of pFcRn promoter luciferase reporter plasmids. (**A**) Schematic diagram of the pFcRn promoter region and luciferase reporter plasmid. (**B**) IPEC-J2 cells were co-transfected with a series of truncated pFcRn promoter constructs (−1740 to +350, F1 to F9) and luciferase reporter vector (pRL-TK-luc), and its luciferase activity was measured. (**C**) IPEC-J2 cells were co-transfected with pFcRn luciferase reporter plasmids and the pRL-TK-luc vector, incubated with TGF-β1 (8 ng/mL) for 12 h, and luciferase activity was quantified; * *p* < 0.05, ** *p* < 0.01.

To investigate whether JNK modulated pFcRn expression by directly binding to the putative c-JUN binding sequences, we performed experiments using the luciferase reporter constructs of the pFcRn promoter plasmids containing c-JUN binding sites. The reporter gene was amplified by PCR using different lengths of the pFcRn promoter region and cloned into the pGL3-basic vector to generate nine luciferase reporter plasmids named F1 to F9 (Figure 3A). The F1 to F9 reporter plasmids were co-transfected into IPEC-J2 cells along with pRL-TK and incubated for 24 h. Next, we measured the basal promoter activity of these plasmids and found that the promoter activity of two luciferase reporter plasmids, F5 and F9, were significantly lower compared to other seven plasmids (Figure 3B). To evaluate the effect of TGF-β1 on pFcRn promoter activity, IPEC-J2 cells were co-transfected with luciferase reporter plasmids (F1 to F9) and pRL-TK for 24 h, and then stimulated by TGF-β1 for 12 h. The quantification of luciferase activity showed that seven luciferase reporter plasmids, F1-4 and F6-8, significantly induced the luciferase activity of the pFcRn promoter in response to TGF-β1 stimulation (Figure 3C). These data suggested that the c-JUN-sensitive region on the pFcRn promoter was located between positions −1246 and −140.

### 3.4. The pFcRn Promoter Contained Three c-JUN Binding Sites as Confirmed by ChIP

**Figure 4:**
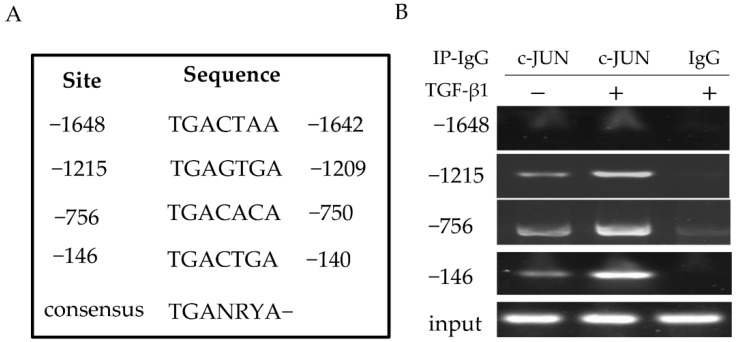
Evaluation of c-JUN binding to porcine pFcRn promoter in vivo. (**A**) The putative c-JUN binding sites in the pFcRn gene are indicated. Numbers show the putative c-JUN binding sites associated with the transcription start site of the pFcRn gene. TGANRYA, N is A or G, Y is any nucleotide, and Y is A, G, or C. (**B**) c-JUN components are present in the pFcRn promoter in vivo in response to TGF-β1 treatment. IPEC-J2 cells were incubated with TGF-β1 (8 ng/mL) for 30 min. The ChIP assay was performed using c-JUN-specific Abs (lane 2). IgG was used as the negative control (lane 3). The DNA fragments were analysed by PCR using the primers specified in Table 1. The ChIP assay was repeated at least three times.

The canonical c-JUN binding sequence is a common 7 bp shared DNA element 5′-TGANRYA-3′ (N could be A or C; R could be A or T; and Y could be A, G, or C). Bioinformatics analysis showed that the pFcRn promoter contained a sequence similar to the c-JUN consensus sequence (Figure 4A). Therefore, we used the ChIP assay to verify that c-JUN was able to bind to these putative c-JUN sequences in cells. First, we stimulated IPEC-J2 cells with TGF-β1 (mock-stimulated cells were used as a control), cross-linked the DNA with bound proteins in situ, and then precipitated DNA-protein complexes with the c-JUN antibody. Next, DNA fragments were analysed, PCR with c-JUN-specific primers (Table 2) generated a band from DNA coprecipitated with c-JUN (−1286, −1128, and −642), while the sequence (−894) failed to generate a band (Figure 4B). In the negative control group, immunoprecipitation using normal mouse IgG did not generate corresponding PCR products. Our data indicated that in IPEC-J2 cells, the c-JUN transcription factor interacted with three c-JUN binding (−1215, −756, −146) sequences in the promoter region of the pFcRn gene.

### 3.5. TGF-β1 Induced pFcRn-Mediated IgG Transcytosis in Polarized IPEC-J2 Cells

**Figure 5:**
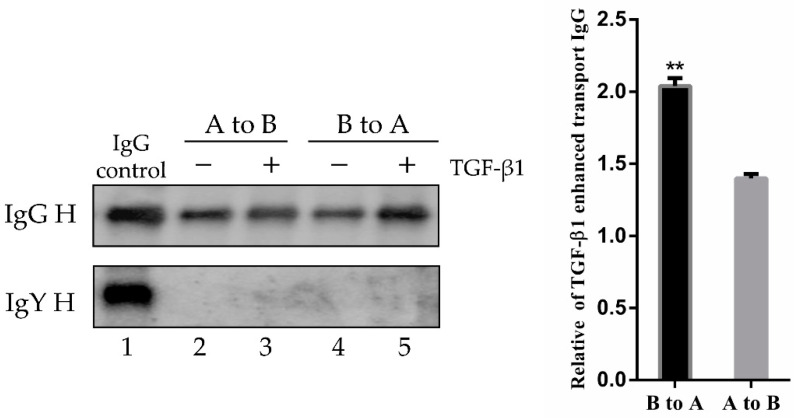
Effect of TGF-β1 stimulation on IgG bidirectional transcytosis. A, apical; B, basolateral. IPEC-J2 cells were cultured on 12-well Transwell plates (0.4 μm pore size) for 6–7 days, until TEER > 1000 Ω/cm^2^. IPEC-J2 cells were incubated with or without TGF-β1 (8 ng/mL) for 12 h. Porcine biotin-IgG or biotin-IgY were added to the apical chamber (lanes 2 and 3) or basolateral chamber (lanes 4 and 5) and incubated for 3 h at 37 °C. Lane 1 corresponds to IgG, ** *p* < 0.01.

The FcRn mediates bidirectional IgG transport in the polarized epithelial cells. Therefore, we hypothesized that TGF-β1 would affect IgG transcytosis in epithelial cells. To test this hypothesis, we used a Transwell system to mimic the porcine mucosal epithelial barrier. The polarized monolayers of IPEC-J2 cells (transepithelial electrical resistance, TEER > 1000 Ω/cm^2^) were treated with TGF-β1 (8 ng/mL) for 12 h. After 12 h, porcine biotin-IgG or chicken biotin-IgY were added to the apical or basolateral side of the IPEC-J2 cell monolayer and incubated for 3 h at 37 °C. The IgG transport of IgY H or IgG H chain to the opposite basolateral or apical side was evaluated by Western blotting (Figure 5, lane 1). Quantification of Western blots showed that IgG transport from the apical to basolateral direction was increased 1.4-fold (Figure 5, lane 3), while transport from the basolateral to the apical side was increased 2-fold by TGF-β1 compared to mock-treated monolayers (Figure 5, lane 5).

## 4. Discussion

Increased FcRn expression can be triggered by the pathogenic invasion of mucosal surfaces, significantly enhancing the defence against pathogens. NF-κB signaling is involved in the upregulation of FcRn via pro-inflammatory factors, such as TNF-α and LPS [18]. TGF-β1 is a cytokine that promotes cell proliferation, extracellular matrix production, and rapid reconstruction of the intestinal epithelial barrier after cell barrier injury [31]. TGEV has been reported to induce FcRn expression through NF-κB signaling and to upregulate TGF-β1 expression in IPEC-J2 cells [28,29,30]. Here, we showed for the first time that TGF-β1 stimulated pFcRn expression in a dose- and time-dependent manner; we also investigated the underlying mechanisms of this upregulation.

FcRn and pIgR have similar regulatory pathways, such as NF-κB and JAK-STAT signaling [16,17,18,19]. p38 MAPK activation is required for increased pIgR/SC expression in epithelial cells cultured in the presence of an activated PMN supernatant [32,33]. Long-term JNK1/2 phosphorylation, in response to TGF-β1 stimulation, plays a critical role in MMP-9 upregulation in rat brain astrocytes (RBA-1 cells) [34]. The activation of the p38 MAPK cascade is important for pIgR/SC expression in the airway [21]. TGF-β1 enhanced JNK phosphorylation, while JNK inhibition reduced the ability to upregulate pFcRn production. However, U0126 and ERK inhibitors did not have an effect on TGF-β1-induced pFcRn production, suggesting that ERK or p38 did not participate in the TGF-β1-induced pFcRn expression.

It has been shown that pFcRn responds to inflammatory stimuli. We identified several transcription factor binding sequences, including binding sequences for AP-1, interferon regulatory factor (IRF1), and p65, as well as three specific binding sites for c-JUN. Several studies have identified NF-κB p65 binding sites in the human and bovine FcRn promoter [18,35]. Interestingly, c-JUN-binding motifs have been previously reported in the human FcRn promoter [36]. Therefore, we decided to validate these predicted binding sites. Here, we present evidence that the JNK transcription factor was able to bind to these potential c-JUN binding sites, as confirmed by the luciferase reporter gene assay and then verified by the sequence-specific binding to the c-JUN site using ChIP. These findings indicate a strong and effective molecular interaction between c-JUN and the selected transcription binding site on the pFcRn promoter. TGF-β1-induced pFcRn is involved in IgG immune complex transcytosis, resulting in antigen uptake via specialized APCs and the activation of the adaptive immune reactions: the antigen is transported to the lamina propria and then taken up by specialized antigen-presenting cells to activate the adaptive immune response. TGF-β1 induced pFcRn-mediated IgG (virus-specific antibody) transcytosis through the mucosal epithelium, hence promoting the anti-viral defence of the host.

In conclusion, this study demonstrated for the first time that TGF-β1 induced pFcRn expression through the JNK/MAPK pathway. By analysing the pFcRn promoter and performing luciferase reporter assays, we showed that the c-JUN binding region is located between −1215 and −140 of the pFcRn promoter. The ChIP assay confirmed three c-JUN binding sites, demonstrating a novel mucosal function of TGF-β1—the upregulation of its receptor-mediated epithelial transport. It has been reported that TGEV infection stimulated TGF-β1 production in IPEC-J2 cells [28], and we found that TGF-β1 induced pFcRn-mediated IgG transcytosis. This result provided a scientific and theoretical basis for the prevention and control of TGEV infection. Several studies have reported that IL-2, IL-4, and IFN-γ, as mucosal immune adjuvants, enhance the body’s mucosal immune response [37]. Since TGF-β1 also enhances antibody and antigen–antibody complex transport via FcRn in vivo, it is possible that TGF-β1 could be used as a mucosal immune enhancer or a novel adjuvant for mucosal immunity.

## Figures and Tables

**Table 1:** Primers used for cloning of pFcRn gene promoter.

Primer	Sequence (5′ to 3′)
pFcRn-luc1-F	GCCGAGCTCAGTGTCCACAATCACATGAGCCA
pFcRn-luc1-R	CCCAAGCTTTCCTCCTCCTCCTCCTCCTCC
pFcRn-luc2-F	GCGAGCTCGACTGAGGTTCTTATCAGGGATGC
pFcRn-luc2-R	CCCAAGCTTTCCTCCTCCTCCTCCTCCTCC
pFcRn-luc3-F	GCGAGCTCCGACCTAGGCGAGGCCAA
pFcRn-luc3-R	CCCAAGCTTTCCTCCTCCTCCTCCTCCTCC
pFcRn-luc4-F	GCGAGCTCGCCGATCTCTAAAGGTGGGG
pFcRn-luc4-R	CCCAAGCTTTCCTCCTCCTCCTCCTCCTCC
pFcRn-luc5-F	GCGAGCTCAGGGATCGCGGCTGCTGT
pFcRn-luc5-R	CCCAAGCTTTCCTCCTCCTCCTCCTCCTCC
pFcRn-luc6-F	GCCGAGCTCAGTGTCCACAATCACATGAGCCA
pFcRn-luc6-R	CCCAAGCTTCTCACAGCAGCCGCGATC
pFcRn-luc7-F	GCCGAGCTCAGTGTCCACAATCACATGAGCCA
pFcRn-luc7-R	CCCAAGCTTCACCTTTAGAGATCGGCGCA
pFcRn-luc8-F	GCCGAGCTCAGTGTCCACAATCACATGAGCCA
pFcRn-luc8-R	CCCAAGCTTTTTTTGGCCTCGCCTAGGTC
pFcRn-luc9-F	GCCGAGCTCAGTGTCCACAATCACATGAGCCA
pFcRn-luc9-R	CCCAAGCTTTCCCTGATAAGAACCTCAGTCGG

**Table 2:** PCR primers for the ChIP assay.

Primer	Sequence (5′ to 3′)
Luc-146-F	TGACGAGGTAAGAAGGGGGC
Luc-146-R	GAGGGTGCCGGCGATCCA
Luc-790-F	TGCTGCGGCTCTGATTACACC
Luc-790-R	TGTGTCAAAACTTCATTTCTTTTTTG
Luc-1215-F	GTGTGTTAAGAACCCGACTGAGG
Luc-1215-R	GCTGAAGCTGTAGATATCAGCCTATAC
Luc-1684-F	TCTTTCTATATATATGCATACATCCTGTTG
Luc-1684-R	TTCTTTTGGATATATAGCTGGGAGTG

## Data Availability

The data that support the findings of this study are available from the corresponding author upon reasonable request.
